# Disease Activity Is Associated with Obesity in Newly Diagnosed Pediatric Patients with Ulcerative Colitis

**DOI:** 10.3390/ijerph192316091

**Published:** 2022-12-01

**Authors:** Orsolya Kadenczki, Antal Dezsofi, Aron Cseh, Daniel Szucs, Noemi Vass, Eva Nemes, Andras Tarnok, Erzsebet Szakos, Ildiko Guthy, Marta Kovacs, Anna Karoliny, Judit Czelecz, Csongor Kiss, Katalin Eszter Müller

**Affiliations:** 1 Department of Pediatrics, Faculty of Medicine, University of Debrecen, 4032 Debrecen, Hungary; 2 1st Department of Pediatrics, Semmelweis University, 1083 Budapest, Hungary; 3 Department of Pediatrics, University of Szeged, 6725 Szeged, Hungary; 4 Department of Pediatrics, Medical School, University of Pécs, 7623 Pécs, Hungary; 5 Borsod Abaúj Zemplén County University Teaching Hospital, University of Miskolc, 3526 Miskolc, Hungary; 6 Szabolcs-Szatmár-Bereg County Hospitals, University Teaching Hospital, 4400 Nyíregyháza, Hungary; 7 Petz Aladar Teaching Hospital, 9024 Győr, Hungary; 8 Heim Pal National Pediatric Institute, 1089 Budapest, Hungary; 9 Bethesda Children’s Hospital, 1146 Budapest, Hungary; 10 Institute of Translation Medicine, Medical School, University of Pécs, 7623 Pécs, Hungary

**Keywords:** obesity, inflammatory bowel disease, children, disease activity index, Crohn’s disease, ulcerative colitis

## Abstract

Malnutrition and inflammatory bowel disease (IBD) are interrelated conditions. Our aim was to assess the prevalence of malnutrition, to compare anthropometric parameters in the evaluation of nutritional status in pediatric IBD, and to investigate the association between anthropometric parameters and disease activity indices (AI). Pediatric patients with newly diagnosed IBD recorded between 2010 and 2016 in the Hungarian Pediatric IBD Registry were included in this cross-sectional study. Body weight, body mass index (BMI), weight-for-height, and ideal body weight percent (IBW%) were analyzed. Pearson linear and non-linear correlations and polynomial regression analyses were performed to assess correlation between nutritional status and AI. *p*-values < 0.05 were considered significant. Anthropometric data of 1027 children with IBD (Crohn’s disease (CD): 699; ulcerative colitis (UC): 328; mean age 13.7 years) were analyzed. IBW% identified more obese patients than BMI both in CD (7.02% vs. 2.28%) and UC (12.17% vs. 5.48%). Significant negative correlation was found among anthropometric parameters and AI in CD. In contrast, polynomial regression analysis revealed a U-shaped correlation curve between IBW% and AI in UC. Our findings show that obesity has a bimodal association with disease activity in pediatric UC. Furthermore, IBW% was more useful to identify obese pediatric patients with IBD.

## 1. Introduction

Inflammatory bowel diseases (IBD), including Crohn’s disease (CD), ulcerative colitis (UC) and inflammatory bowel disease-unclassified (IBD-U), are chronic immune-mediated, relapsing-remitting disorders of the gastrointestinal tract. Incidence of pediatric IBD is growing worldwide, including in Hungary [1]. Malnutrition is frequently observed in pediatric patients with IBD. About two-thirds of patients are undernourished at the time of diagnosis, and they have an increased risk for morbidity and mortality [2,3]. Poor nutritional status may result in linear growth retardation that is considered as an extraintestinal manifestation in pediatric IBD [4]. Prevalence of moderate and severe underweight among children and adolescents in the general population is as low in Hungary as in other Western and Central European countries [5]. A recent WHO-initiated survey of 6-to-9-year-old children found 3.8% of Hungarian boys and 2.3% of Hungarian girls thin [6]. The prevalence of Hungarian children between the age of 1 and 5 years with body weight below 3rd centile varied between 1.70% and 3.16% in the age groups 1, 3, and 5 years of age, respectively, and in three consecutive years from 2013 to 2015 [7]. The relationship between undernutrition and IBD is well known. Severe undernutrition is one of the extraintestinal manifestations of Crohn’s disease, so we focused on the less well characterized associations of obesity and IBD.

The other side of the malnutrition spectrum, obesity, is an existing socio-epidemiologic problem in all ages, particularly in the Western world. Growing prevalence of childhood obesity is a major public health concern of the century. In 2016, 124 million children between the ages of 5 and 19 years were considered to be obese worldwide [5]. The prevalence of childhood obesity in the USA is 18.5% [8]. In Europe, the prevalence of obesity ranges from 10% in Northern Europe to more than 40% in Southern Europe, affecting 2.1–19.8% of girls and 1.8–19.9% of boys below the age of 10 years in different countries [9]. The rate of obesity among adolescents has also increased between 2010 and 2018 in European countries, mainly in Southern Europe [10]. In Hungary, a population-based survey found that 6.6% of children and adolescents were obese [11], Factors associated with obesity among children and adolescents have not been extensively studied in Hungary. Similar to Cyprus and Italy, and in contrast to five other European countries, no association was found between parental socio-economic status and overweight of children in Hungary according to results of the IDEFIC consortium [12].

The role of obesity in the etiology of IBD is not fully understood, especially in the pediatric population. Several clinical observations found an association between obesity and IBD in adults. Young women were suggested to have an increased risk for developing CD. Unfavorable clinical course of the disease, such as need for biologic treatment, loss of response to infliximab, perianal manifestation, earlier time to first surgery, and decreased quality of life were noted [13]. In contrast, the EPIC study, investigating males and elderly patients in addition to young females, did not find any association of obesity with disease location and phenotypic disease expression [14].

Data on potential associations between obesity and pediatric IBD are scanty. In contrast to the results of adult cohorts, patients with UC were more commonly obese compared to patients with CD. North American cohorts with newly diagnosed IBD found 9–10% of pediatric patients with CD and 20–34% of patients with UC overweight [15]. In a Polish retrospective study, 1.9% of patients with CD and 8.4% with UC were obese at diagnosis [16]. A study from Israel found that pediatric patients with IBD both in the lower and higher ranges of the body mass index (BMI) spectrum had higher disease activity, more frequent disease exacerbations and more frequent need for anti-TNF-α therapy [17].

Nutritional condition in the pediatric population has been characterized most frequently by BMI [16,17,18]. The recent guidance of the European Society of Pediatric Gastroenterology, Hepatology and Nutrition (ESPHGAN) also suggested to use weight, height, and BMI Z-scores for the evaluation of the nutritional status in pediatric patients with IBD [19]. However, cut-off values defining obesity differ between individual trials and publications. Thus, studies may not be comparable. For example, in some studies, BMI was considered abnormal above +2 Z-score values, whereas in some others, BMI was considered elevated above the 75th, the 85th, or the 95th centile values [17,18,20]. Since Hungarian reference curves do not list 85th and 95th centile values, a comparison of nutritional status and risk assignment of Hungarian pediatric patients with IBD based on these cut-off values can be done only by approximation.

Body weight (BW), weight-for-height (WFH) percentiles and Z-scores, and ideal body weight percent (IBW%) are less frequently used anthropometric tools among children with IBD to characterize nutritional status. However, in addition to BMI, some of these indices were successfully used to characterize nutritional status of children with cancer, cystic fibrosis, and chronic liver disease [21,22,23]. IBW has an important role in the accurate definition of chemotherapeutic drug dosages among obese children [24]. IBW% was found more sensitive than BMI to characterize nutritional status in children with cancer [21].

Prior to the present survey, no data on the prevalence of obesity in Hungarian children and adolescents with IBD were published. Moreover, no study has been aimed at investigating a potential association between obesity and disease activity of pediatric IBD so far. The findings of this research may open a new path for further analysis of possible interactions between obesity and IBD in pediatric patients.

Therefore, the aims of the present study were (i) to assess the prevalence of malnutrition among children with newly diagnosed IBD in Hungary with a special emphasis on the rate of obesity; (ii) to implement a complete set of anthropometric parameters, such as BW, WFH Z-scores, and IBW % in addition to BMI; (iii) to compare patients by disease type (CD vs. UC) based on their nutritional status; and (iv) to analyze possible associations between nutritional status of patients as determined by anthropometric parameters and disease activity at the time of diagnosis.

## 2. Patients and Methods

### 2.1. Study Design

We conducted a cross-sectional analysis including patients registered in the prospective, nationwide Hungarian Pediatric IBD Registry (HUPIR) [25,26]. Diagnosis of IBD was based on the Porto criteria [27]. Exclusion criteria were age at diagnosis older than 18 years, missing information on ileocolonoscopy and ileocolonic histology, a diagnostic workup without endoscopic, histologic, and radiologic abnormalities, and patients without informed consent. In the registry, age, gender, weight, and height were recorded. The survey obtained the data anonymously. HUPIR fulfils two functions: it is used as a database and data serve research purposes [25]. Therefore, informed consent covered both consenting to the patients’ data to be registered and to use these data for the purposes of further analyses.

Pediatric patients between 0 and 18 years diagnosed with CD or UC between 2010 and 2016 were included in this cross-sectional study. During this period, 1027 children were registered, 699 patients with CD and 328 patients with UC. Fifty-four patients with IBD-U were excluded in a second step because the low number did not allow proper statistical evaluation of this subpopulation. Supplementary Figure S1 illustrates the flowchart of patients recruited in the study.

The study was approved by the Scientific Research Ethical Committee of the Medical Research Council of Hungary (10434/2012/EKU 175/PI/12) and was performed according to the 2008 Declaration of Helsinki. Written informed consent was obtained from legal guardians of participating patients.

### 2.2. Assessment of Nutritional Status

Nutritional status of patients was determined at diagnosis by BMI Z-scores and other anthropometric parameters based on the weight and height of patients according to the guidance of the ESPHGAN position paper [19]. BW (in kg) and height (in cm) of patients were recorded in the HUPIR database at the time of diagnosis. BW, WFH, and BMI (kg/m^2^) were calculated together with standard deviation scores (SDS; Z-score) based on the reference values of the Hungarian Longitudinal Child Growth Survey [28]. IBW% was calculated as actual BW*100/50th percentile WFH at the same time. Undernutrition was defined as BW or WFH or BMI Z-score < −2.0, obesity was defined as Z-score > 2.0, respectively, based on WHO criteria [29]. If IBW% was <90%, patient was considered undernourished, whereas IBW% >120% was considered obese. Degree of undernutrition was interpreted by IBW%, where severe undernutrition was defined as IBW% < 70%, moderate as 70–80%, and mild as 80–90% [30].

### 2.3. Disease Activity of CD and UC at Diagnosis

Disease activity was determined using validated clinical activity indices (AI): the Pediatric Ulcerative Colitis Activity index (PUCAI) and the Pediatric Crohn’s Disease Activity Index (PCDAI) [31,32]. The range of PUCAI score varies between 0 and 85, and the range of PCDAI score varies between 0 and 100, where higher values (>10) are associated with more severe disease. PUCAI classifies disease activity as inactive with <10 points, mild between 10 and 35 points, moderate between 35 and 65 points, and severe exceeding 65 points. PCDAI categories were as follows: inactive disease <10 points, mild between 11 and 30 points, moderate to severe >30 points.

### 2.4. Statistical Analysis

Categorical variables between patients with CD and UC were assessed by chi-square or Fisher exact test. Kolgomorov–Smirnov test was used to investigate normality of continuous variables. Quantitative variables were compared by means of Kruskal–Wallis or Mann–Whitney test. Pearson correlation was performed to assess the correlation between nutritional status and AI. Firstly, linear correlation analysis was performed to reveal the interaction between nutritional status and disease activity. Secondly, quadratic and cubic curve modeling and polynomial regression analysis were carried out to characterize non-linear correlation between anthropometric data and disease activity. *p*-Values less than 0.05 were considered significant. Statistical analysis was performed using IBM SPSS Statistics 26.0.0.0.

## 3. Results

### 3.1. Patient Characteristics

A total of 1027 children were included in this study, 699 with CD (66%) and 328 (32%) with UC. Among patients with CD, the median age at diagnosis was 14.4 years (mean 13.7 years, range between 1.1 and 18.0 years). Among patients with UC, the median age was 13.4 years (mean 12.6 years, range between 1.6 and 18.0 years). Four-hundred and seven (41.77%) patients with CD and 158 (48.17%) patients with UC were females. According to disease AI, there were 343 (50.66%) mild, 152 (22.90%) moderate, and 149 (22.00%) severe cases of CD, and 143 (45.68%) mild, 140 (44.72%) moderate, and 30 (9.60%) severe cases of UC. There were only 30 children with an AI indicating inactive disease at diagnosis although Porto criteria fulfilled the presence of IBD (Table 1). Lack of registered disease activity indices in the cases of 37 patients in the database decreased the total number of analyzed patients to 990.

Hungarian reference curves do not contain data above the height of 184 cm in boys and 175 cm in girls, so BMI, WFH, and IBW% calculation was not possible in 42 exceedingly tall adolescent patients. Therefore, analyses influenced by body height were performed only in a restricted number (N = 985) of patients. This data loss, however, did not influence statistical analysis because of the high number of patients with sufficient data (Figure S1).

### 3.2. Prevalence of Nutritional Status Deviations among Pediatric Patients with IBD in Hungary

Nutritional status of Hungarian pediatric patients with IBD, as characterized by BW, WFH, BMI Z-scores, and IBW% are summarized in Table 2. Mean and median values of BW, BMI, WFH Z-scores, and IBW% are demonstrated in Supplementary Table S1.

**Figure 1:**
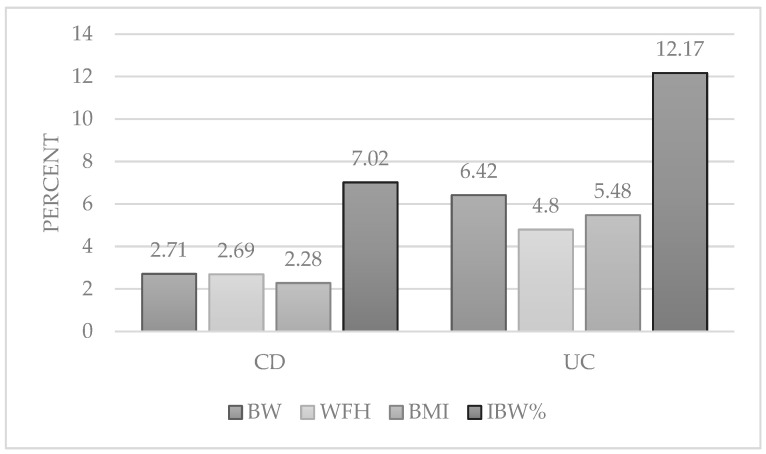
Prevalence of obesity among patients with CD and UC based on different anthropometric parameters. CD: Crohn’s disease; UC: ulcerative colitis; BW: body weight; WFH: weight-for-height; BMI: body mass index; IBW%: ideal body weight percent.

Most patients were well-nourished, irrespective of diagnosis (CD or UC) and of the investigated anthropometric parameters. Obese and undernourished patients represented a minority of the patient population without significant difference between the frequencies of these two types of nutritional status deviations. There was a tendency for increased prevalence of obesity among patients with UC as categorized by all anthropometric parameters, but the difference was not significant compared to the rate of obesity among patients with CD; 2.71, 2.28, 2.69, and 7.02 percent of patients with CD were obese calculated by BW, BMI, WFH Z-score, and IBW%, respectively, while 6.42, 5.48, 4.80, and 12.17% were obese in the UC group according to the above parameters (Figure 1). When analyzing the other end of the spectrum of nutritional status, more patients with CD were undernourished, as defined by all parameters, compared to patients with UC, but the difference was not significant between the two groups.

### 3.3. Assosiation between Nutritional Status Deviations and Disease Activity among Pediatric Patients with IBD in Hungary

At the time of diagnosis, the median PCDAI of patients with CD was 30 (mean: 30.60 ± 15.03; range: 0–87.5), and the median PUCAI in UC group of patients was 35 (mean 38.56 ± 19.02; range 5–85). Supplementary Table S2 shows mean and median activity indices of patients with CD and UC categorized according to different groups of nutritional status.

Disease AIs in patients with different nutritional status defined by BW, WFH, BMI Z-score, and IBW% were analyzed. In the group of patients with CD, AI was significantly higher in undernourished than in well-nourished and obese patients regardless of the anthropometric parameter used. PCDAI showed a further significant decrease in obese patients vs. well-nourished patients (Table 3).

In contrast, in patients with UC, only estimations based on WFH and BMI tools exhibited significant differences between the disease activity indices of subgroups according to nutritional status. Similar to patients with CD, disease activity of undernourished patients was significantly higher than that of well-nourished or obese patients. However, no significant differences in PUCAI among well-nourished and obese patients were observed.

Linear regression analysis did not reveal any significant correlation between anthropometric measures and AIs when analyzing the total population of patients with either CD or UC. As our previous results suggested that a more complex correlation may exist between AIs and nutritional status, we divided the total populations of patients with CD and UC into two groups. Patients below and above the median values of the applied anthropometric parameters were analyzed separately by linear regression. Supplementary Table S3 summarizes correlations between disease activity indices and nutritional status.

A significant negative correlation was found between PCDAI and nutritional status investigating with each anthropometric parameter in patients with CD below the median values (BW Z-score R: −0.148, B: −7.440, *p*: 0.006, WFH Z-score R: −0.221, B: −7.080, *p*: 0.000, BMI Z-score R: −0.314, B: −10.745, *p*: 0.000, IBW% R: −0.194, B: −0.465, *p*: 0.000). In contrast, among patients with CD characterized by anthropometric parameters exceeding the median values, there was not any significant correlation.

In the subgroup of patients with UC characterized by anthropometric values below the median values, only the WFH (R: −0.196, B: −6.955, *p*: 0.002) and IBW% (R: −0.174, B: −0.537, *p*: 0.033) tools were able to reproduce the same significant negative correlation with disease activity as found in patients with CD. In the subgroup of patients with UC above the median value of IBW%, an opposite, significant positive correlation with disease activity was observed (R: 0.166, B: 0.171, *p*: 0.044). A U-shaped correlation can be observed between disease activity and nutritional status.

**Figure 2:**
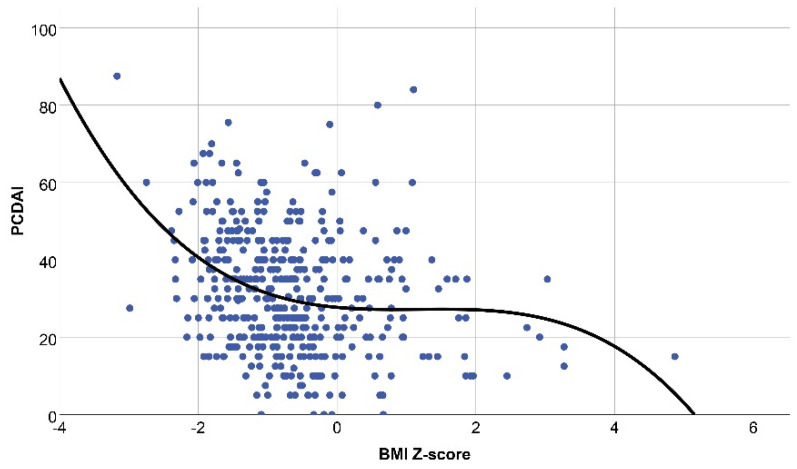
Correlation between body mass index Z-score and disease activity in Crohn’s disease by polynomial regression analysis (R = 0.22, *p* = 0.000). PCDAI: pediatric Crohn’s disease activity index; BMI: body mass index.

Performing further analysis, polynomial regression was introduced to uncover the correlation of disease activity and nutritional status. In patients with CD, all investigated parameters showed declining significant cubic correlation with disease activity with R values of 0.22, 0.22, 0.22, and 0.23 (*p* = 0.000) for BW, WFH, BMI, and IBW%, respectively (Figure 2).

**Figure 3:**
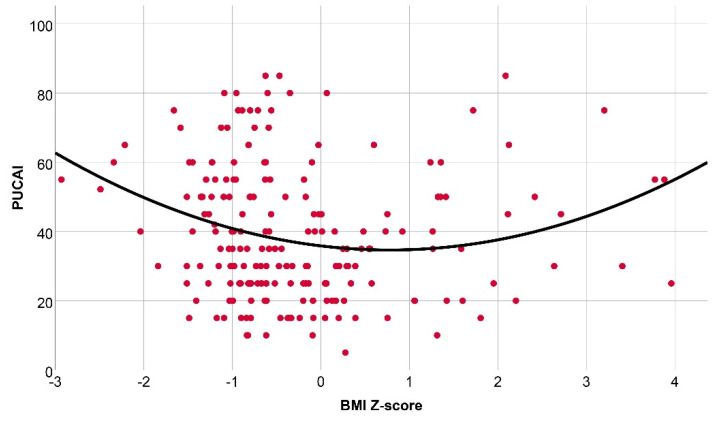
Correlation between body mass index Z-score and disease activity in UC by polynomial regression analysis (R = 0.20, *p* = 0.006). PUCAI: pediatric ulcerative colitis activity index; BMI: body mass index.

In contrast, in the whole population of patients with UC we observed a characteristic U-shaped curve when investigating PUCAI in relation to nutritional status by polynomial analysis. All nutritional parameters such as BW, WFH, BMI, and IBW% correlated significantly in a quadratic manner with disease activity with R values of 0.16 (*p* = 0.019), 0.19 (*p* = 0.004), 0.20 (*p* = 0.019), and 0.19 (*p* = 0.006), respectively. These results suggest a bimodal association between anthropometrical data and disease activity in patients with UC (Figure 3).

## 4. Discussion

The present cross-sectional study is the first one in Hungary that assessed the nutritional status of newly diagnosed pediatric patients with IBD focusing on the prevalence of obesity and on the relationship between nutritional status and disease activity. The data from this epidemiologic study were collected from HUPIR, a nationwide, prospective registry of pediatric patients with IBD. The main result of this investigation is that prevalence of obesity among IBD patients is similar to that of the general Hungarian pediatric population, and obesity has a bimodal association with disease activity in patients with UC.

Prevalence of both obesity and IBD are growing worldwide among adults and children [29,33]. As reviewed by Singh et al., there is an association between premorbid obesity and the risk of developing CD, but not UC, among adults [34]. A similar trend has also been observed in the pediatric IBD population, with the difference that the prevalence of obesity was higher among children and adolescents with UC rather than CD [15].

Analyzing patient data of HUPIR in our present study indicated that undernutrition was less frequent among children both with CD and UC than found by other investigators from different countries [2,3]. The prevalence of obesity classified by BMI was in the same range among patients with IBD (2.28% in CD and 5.48% in UC) as among the general Hungarian childhood population (5.50% among boys and 7.50% among girls) [11].

In Croatia, Sila et al. did not find any obese individuals among pediatric patients either with CD or with UC. The prevalence of obesity among healthy children was 8.20% [35]. In contrast, in a Polish multicenter, retrospective study, 1.90% of pediatric patients with CD and 8.47% with UC were obese as defined by centile values (≥95th centile). In parallel with this observation, 18.60% of healthy pediatric males and 14.50% of females were obese in Poland [16]. In recent studies from North America, the prevalence of obese pediatric patients with CD is about four times, and that of patients with UC is three times higher than in Hungary [20,36]. Differences between the North American and the Hungarian pediatric populations could be attributed to the fact of differences in dietary habits as these countries are geographically, traditionally, and socio-economically far away from each other. It seems, the prevalence of obesity in pediatric patients with IBD varies across countries even within the same geographic region, such as Hungarian, Croatian, and Polish data characterizing Central Europe and even more between countries in different continents, as pointed out by comparing Hungarian and US data.

The nutritional status of patients was defined by anthropometric parameters according the most resent ESPHGAN position paper [19]. In our study most of the children had BM, WFH, BMI, Z-scores, and IBW% values within the normal ranges. Prevalence of undernutrition was higher than obesity among patients with CD. Undernutrition is a well-known extraintestinal manifestation of pediatric CD since the intestinal pathology and the inflammatory response associated with CD result in weight loss. This explains that the activity of the disease at the time of diagnosis was more pronounced among undernourished children. Obesity, on the other hand, was more frequent among patients with UC than with CD. Median disease AIs of undernourished and obese patients with UC were similarly high in contrast to well-nourished patients with UC. Polynomial regression analysis indicated—for the first time—an association between obesity as determined by anthropometrical indices and disease activity in pediatric UC, suggesting that obesity may have a negative effect in this disease. The association was particularly prominent when nutritional status was determined by IBW%.

Our results support the concept that obesity, as a proinflammatory condition by itself, may contribute to the aggravation of disease activity in UC. Moreover, the fact of being obese may represent a risk factor for developing UC [15]. Hidden, intra-abdominal accumulation of fatty tissue is not typical in UC [37]. In contrast, the presence of mesenterial creeping fat is a known phenomenon in patients with CD, even in the well-nourished and undernourished subpopulation [38]. Therefore, under-, and well-nourished patients may also suffer from creeping fat-induced inflammation [39]. The fact that obesity is a proinflammatory condition may be of special importance in countries such as Hungary, where the ratio of obesity in the general pediatric population is considerably high. Obesity represents the accumulation of abnormal or excessive fat tissue, resulting in weight gain. In CD, where normal weight or undernutrition is more common than obesity, fat accumulation does not necessarily coincide with weight gain. Elevated expression of adiponectin was found in creeping-fat of patients with active CD [40]. Secretory adipose tissue changes in CD may be observed most frequently in the mesenteric adipose tissue (creeping fat) which, in addition to local changes, may exert systemic effects as it produces soluble inflammatory mediators, such as C-reactive protein (CRP) [41]. An elevation in CRP level increases PCDAI, which is the indicator of disease activity. In contrast, PUCAI does not include data on inflammatory biomarkers, suggesting that inflammation is not a factor of disease activity in pediatric UC. Although evaluation of CRP levels was not included in this study—because of the lack sufficient data—the observed association between obesity and high PUCAI score at diagnosis may suggest a so far unrecognized relationship between obesity, inflammation, and pediatric UC.

A clear correlation between disease activity and anthropometric parameters was found with using IBW% as a tool to determine nutritional status, not with the more generally accepted BMI, which is the recommended and most widely used index to define obesity among the general population and in pediatric patients with IBD [19,42,43,44]. The use of anthropometric parameters beyond BMI has been less commonly used but may be strongly recommended for some patient groups [21,22,23]. In this study, we assessed nutritional status based on BW, WFH Z-scores, and IBW% in addition to BMI Z-scores and found that the acuteness of these parameters was similar, except IBW%. For the calculation of IBW%, we used the 50th centile WFH value of healthy children. In the investigated population, more children proved to be obese when determined by IBW% compared to BW, WFH, and BMI Z-scores. IBW% identified 7.02% of patients with CD as obese, compared to 2.71%, 2.28%, and 2.69% assessed by BW, BMI, and WFH Z- scores, respectively. Among patients with UC, the rates of obesity were 12.17% by IBW% and 6.42%, 5.48%, and 4.80% by BW, BMI, and WFH Z-scores, respectively. This may be clinically relevant because defining obesity by IBW% does not depend on arbitrary cut-off values, in contrast to BMI.

Detecting obesity as early as possible may be beneficial in influencing long-term outcomes in pediatric IBD. Determining IBW% in addition to the recommended BMI Z-score at regular patient visits may be helpful in governing dietary treatment and avoiding complications [19]. Recent studies found that not only undernutrition but also obesity, as determined by BMI, increased the risk of hospitalization and disease exacerbation in childhood IBD [43]. Von Graffenried et al. found that overweight and obesity were more prevalent among children with UC and were more frequently associated with perianal abscesses and surgery for this purpose among children with CD [45]. Although we have not studied disease course, including outcome of biological treatment if indicated, especially in patients with UC, or surgery in patients with CD, determining IBW% in addition to BMI may be a useful tool to predict complications in pediatric patients in IBD.

BMI or IBW% determination alone, however, may not be sufficient to assess nutritional status. Examination of body fat distribution e.g., by whole body impedance analysis (BIA), computer tomography (CT), or magnetic resonance imaging (MRI) might have an important added value, as the visceral adipose tissue of non-obese patients with low BMI may also function as proinflammatory tissues [46]. Moreover, studying gut microbiota in patients with IBD may also have an important added value since a possible exciting relationship was revealed recently between obesity and changes is gut microbiota in patients with CD and UC [47]. The link between IBD and Western-style diet and changes in the gut microbiota was recently published [48].

The present study has a few limitations. One of the limitations is the lack of analyzing body composition with BIA, CT, or MRI. Anthropometric measures for evaluating subcutaneous adipose tissue accumulation, such as mid-upper arm circumference and skinfold thickness, were not registered in HUPIR. A further limitation of our study is that we did not investigate the effect of dietary habits of patients on nutritional status and disease activity. However, the major limitation is the cross-sectional nature of this study. Because of the lack of patient follow-up, we cannot define properly possible harmful effects of obesity at diagnosis of pediatric patients with IBD. Further prospective studies should be designed to assess the role of obesity in de novo pediatric IBD on disease outcome markers. Moreover, biomarkers of inflammation and of body composition should be involved in further investigations.

## 5. Conclusions

Obesity, in particular, if determined by IBW%, proved an important risk factor of disease activity of UC in the Hungarian pediatric patient population. IBW% is a less commonly used anthropometric parameter to determine nutritional status versus the more frequently used BMI. The study revealed that IBW% was a valuable method for assessing nutritional status in children with IBD, identifying more patients with improper nutritional status than BMI. Given that not only undernutrition but also obesity affects adversely the disease activity of IBD, it is essential to detect children with overweight as soon as possible and to take preventive actions.

## Figures and Tables

**Table 1:** Patient characteristics at diagnosis.

	CD (N = 699) 66.06%	UC (N = 328) 31.94%
Age, mean (±SD) (years)	13.7 (±3.19)	12.6 (±3.73)
Age, median (range) (years)	14.4 (1.1–18.0)	13.4 (1.6–18.0)
Sex		
Male, n (%)	407 (58.22)	170 (51.82)
Female, n (%)	292 (41.77)	158 (48.17)
PCDAI/PUCAI	(N = 677)	(N = 313)
Inactive disease	30 (4.44)	0
Mild n (%)	343 (50.66)	143 (45.68)
Moderate n (%)	155 (22.90)	140 (44.72)
Severe n (%)	149 (22.00)	30 (9.60)

CD: Crohn’s disease; UC: ulcerative colitis; SD: standard deviation; N: number of patients.

**Table 2:** Nutritional status of patients with CD and UC by different anthropometric parameters.

Anthropometric Parameter	CD N (%)	UC N (%)
Body weight	699 (100)	328 (100)
Undernourished (Z-score < −2)	36 (5.16)	2 (0.60)
Normal (−2 < Z-score < 2)	644 (92.13)	305 (92.98)
Obese (Z-score > 2)	19 (2.71)	21 (6.42)
Body mass index	699 (100%)	328 (100%)
Undernourished (Z-score < −2)	19 (2.70)	6 (1.83)
Normal (−2< Z-score < 2)	664 (94.99)	304 (92.67)
Obese (Z-score > 2)	16 (2.28)	18 (5.48)
Weight-for-height	669 (100%)	312 (100%)
Undernourished (Z-score < −2)	29 (4.34)	7 (2.25)
Normal (−2 < Z-score < 2)	622 (92.97)	290 (92.95)
Obese (Z-score > 2)	18 (2.69)	15 (4.80)
Ideal body weight percent	669 (100%)	312 (100%)
<70	22 (3.28)	2 (0.64)
70–80	127 (18.90)	24 (7.70)
80–90	188 (28.30)	79 (25.33)
90–110	236 (35.20)	141 (45.19)
110–120	49 (7.32)	28 (8.97)
>120	47 (7.02)	38 (12.17)

CD: Crohn’s disease; UC: ulcerative colitis; N: number of patients.

**Table 3:** Differences between the mean values of activity indices in the three groups of patients according to nutritional status.

Anthropometric Parameter		AI of Undernourished vs. Normal	*p*	AI of Normal vs. Obese	*p*	AI of Undernourished vs. Obese	*p*
BW	CD	38.63 vs. 30.5	0.06	30.50 vs. 18.67	<0.01	38.63 vs. 18.67	<0.01
WFH		45.00 vs. 31.13	<0.01	31.13 vs. 18.92	0.022	45.00 vs. 18.92	<0.01
BMI		42.15 vs. 30.48	0.002	30.48 vs. 22.94	0.019	42.15 vs. 22.94	<.0.01
IBW%		43.29 vs. 30.60	<0.01	30.60 vs. 27.76	0.01	43.29 vs. 27.76	<0.01
BW	UC	57.50 vs. 38.12	0.330	38.12 vs. 44.25	0.203	57.50 vs. 44.25	0.554
WFH		54.44 vs. 38.32	0.015	38.32 vs. 45.76	0.180	54..4 vs. 45.76	0.267
BMI		54.60 vs. 37.63	0.032	37.63 vs. 44.66	0.211	54.60 vs. 44.66	0.336
IBW%		52.50 vs. 37.74	0.213	37.74 vs. 41.66	0.243	52.50 vs. 41.66	0.444

AI: activity index; BW: body weight; WFH: weight-for-height; BMI: body mass index; IBW%: ideal body weight percent; *p*: *p*-value (level of significance); N: number of patients; SD: standard deviation.

## Data Availability

Data of HUPIR support reported results, but these data are not public.

## Supplementary Materials

The following supporting information can be downloaded at: https://www.mdpi.com/article/10.3390/ijerph192316091/s1, Figure S1: Flowchart of patients; Table S1: Mean and median value of body weight, BW, BMI, WFH Z-scores, and IBW% in patients with CD and UC.; Table S2: Mean and median disease activity indices in patients with CD and UC at the time of diagnosis according to different nutritional status; Table S3. Associations between disease activity indices and nutritional status in patients with CD and UC (Pearson analysis).

## References

[1] Ng S.C., Shi H.Y., Hamidi N., Underwood F.E., Tang W., Benchimol E.I., Panaccione R., Ghosh S., Wu J.C., Chan F.K. (2017). Worldwide incidence and prevalence of inflammatory bowel disease in the 21st century: A systematic review of population-based studies. Lancet.

[2] Sawczenko A., Sandhu B., Logan R., Jenkins H., Taylor C., Mian S., Lynn R.M. (2001). Prospective survey of childhood inflammatory bowel disease in the British Isles. Lancet.

[3] Nguyen G.C., Munsell M., Harris M.L. (2008). Nationwide prevalence and prognostic significance of clinically diagnosable protein-calorie malnutrition in hospitalized inflammatory bowel disease patients. Inflamm. Bowel Dis..

[4] Rosen M.J., Dhawan A., Saeed S.A. (2015). Inflammatory Bowel Disease in Children and Adolescents. JAMA Pediatr..

[5] NCD Risk Factor Collaboration (NCD-RisC) (2017). Worldwide trends in body-mass index, underweight, overweight, and obesity from 1975 to 2016: A pooled analysis of 2416 population-based measurement studies in 128.9 million children, adolescents, and adults. Lancet.

[6] Spinelli A., Buoncristiano M., Nardone P., Starc G., Hejgaard T., Júlíusson P.B., Fismen A.S., Weghuber D., Musić Milanović S., García-Solano M. (2021). Thinness, overweight, and obesity in 6- to 9-year-old children from 36 countries: The World Health Organization European Childhood Obesity Surveillance Initiative—COSI 2015–2017. Obes. Rev. Off. J. Int. Assoc. Study Obes..

[7] Kádár M., Szőllősi G., Molnár S., Szabó L. (2019). The incidence of malnutrition between 1 and 5 years of age on the basis of the preventive primary care data. Dev. Health Sci. DHS.

[8] Hales C.M., Carroll M.D., Fryar C.D., Ogden C.L. (2017). Prevalence of Obesity among Adults and Youth: United States, 2015–2016.

[9] Ahrens W., Pigeot I., Pohlabeln H., De Henauw S., Lissner L., Molnár D., Moreno L.A., Tornaritis M., Veidebaum T., Siani A. (2014). Prevalence of overweight and obesity in European children below the age of 10. Int. J. Obes..

[10] OEU (2020). Overweight and obesity among children and adolescents. Health at a Glance: Europe 2020: State of Health in the EU Cycle.

[11] Jakab A.E., Hidvégi E.V., Illyés M., Cziráki A., Bereczki C. (2018). Prevalence of Overweight and Obesity in Hungarian Children and Adolescents. Ann. Nutr. Metab..

[12] Bammann K., Gwozdz W., Lanfer A., Barba G., De Henauw S., Eiben G., Fernandez-Alvira J.M., Kovács E., Lissner L., Moreno L.A. (2013). Socioeconomic factors and childhood overweight in Europe: Results from the multi-centre IDEFICS study. Pediatr. Obes..

[13] Khalili H., Ananthakrishnan A.N., Konijeti G.G., Higuchi L.M., Fuchs C.S., Richter J.M., Chan A.T. (2015). Measures of obesity and risk of Crohn’s disease and ulcerative colitis. Inflamm. Bowel Dis..

[14] Chan S.S.M., Luben R., Olsen A., Tjonneland A., Kaaks R., Teucher B., Lindgren S., Grip O., Key T., Crowe F. (2013). Body mass index and the risk for Crohn’s disease and ulcerative colitis: Data from a European Prospective Cohort Study (The IBD in EPIC Study). Am. J. Gastroenterol..

[15] Kugathasan S., Nebel J., Skelton J.A., Markowitz J., Keljo D., Rosh J., Leleiko N., Mack D., Griffiths A., Bousvaros A. (2007). Body mass index in children with newly diagnosed inflammatory bowel disease: Observations from two multicenter North American inception cohorts. J. Pediatr..

[16] Pituch-Zdanowska A., Banaszkiewicz A., Dziekiewicz M., Łazowska-Przeorek I., Gawrońska A., Kowalska-Duplaga K., Iwańczak B., Klincewicz B., Grzybowska-Chlebowczyk U., Walkowiak J. (2016). Overweight and obesity in children with newly diagnosed inflammatory bowel disease. Adv. Med. Sci..

[17] Yerushalmy-Feler A., Ben-Tov A., Weintraub Y., Amir A., Galai T., Moran-Lev H., Cohen S. (2018). High and low body mass index may predict severe disease course in children with inflammatory bowel disease. Scand. J. Gastroenterol..

[18] Long M.D., Crandall W.V., Leibowitz I.H., Duffy L., Del Rosario F., Kim S.C., Integlia M.J., Berman J., Grunow J., Colletti R.B. (2011). Prevalence and epidemiology of overweight and obesity in children with inflammatory bowel disease. Inflamm. Bowel Dis..

[19] Miele E., Shamir R., Aloi M., Assa A., Braegger C., Bronsky J., de Ridder L., Escher J.C., Hojsak I., Kolaček S. (2018). Nutrition in Pediatric Inflammatory Bowel Disease: A Position Paper on Behalf of the Porto Inflammatory Bowel Disease Group of the European Society of Pediatric Gastroenterology, Hepatology and Nutrition. J. Pediatr. Gastroenterol. Nutr..

[20] Medina Carbonell F.R., Choyudhry Chandan O. (2020). Body Mass Index at Presentation of Inflammatory Bowel Disease in Children. Pediatr. Gastroenterol. Hepatol. Nutr..

[21] Kadenczki O., Nagy A.C., Kiss C. (2021). Prevalence of Undernutrition and Effect of Body Weight Loss on Survival among Pediatric Cancer Patients in Northeastern Hungary. Int. J. Environ. Res. Public Health.

[22] Sullivan J.S., Mascarenhas M.R. (2017). Nutrition: Prevention and management of nutritional failure in Cystic Fibrosis. J. Cyst. Fibros..

[23] Yang C.H., Perumpail B.J., Yoo E.R., Ahmed A., Kerner J.A. (2017). Nutritional Needs and Support for Children with Chronic Liver Disease. Nutrients.

[24] RRoss E.L., Heizer J., Mixon M.A., Jorgensen J., Valdez C.A., Czaja A.S., Reiter P.D. (2015). Development of recommendations for dosing of commonly prescribed medications in critically ill obese children. Am. J. Health Syst. Pharm..

[25] Müller K.E., Lakatos P.L., Arató A., Kovács J.B., Várkonyi A., Szűcs D., Szakos E., Sólyom E., Kovács M., Polgár M. (2013). Incidence, Paris classification, and follow-up in a nationwide incident cohort of pediatric patients with inflammatory bowel disease. J. Pediatr. Gastroenterol. Nutr..

[26] Müller K., Veres G. (2013). A gyermekkori gyulladásos bélbetegségek hazai regiszterének (HUPIR) eredményei és az 5 éves nyomonkövetés hatása a diagnosztikai gyakorlatra. Magy. Belorv. Archivum..

[27] IBD Working Group of the European Society for Paediatric Gastroenterology, Hepatology and Nutrition (2005). Inflammatory bowel disease in children and adolescents: Recommendations for diagnosis—The Porto criteria. J. Pediatr. Gastroenterol. Nutr..

[28] Joubert K.G. (2016). The Hungarian Longitudinal Growth Study: From Birth to the Age of 18 Years.

[29] World Obesity Federation. https://www.worldobesity.org.

[30] Borowitz D., Baker R.D., Stallings V. (2002). Consensus report on nutrition for pediatric patients with cystic fibrosis. J. Pediatr. Gastroenterol. Nutr..

[31] Turner D., Otley A.R., Mack D., Hyams J., De Bruijne J., Uusoue K., Walters T.D., Zachos M., Mamula P., Beaton D.E. (2007). Development, validation, and evaluation of a pediatric ulcerative colitis activity index: A prospective multicenter study. Gastroenterology.

[32] Hyams J.S., Ferry G.D., Mandel F.S., Gryboski J.D., Kibort P.M., Kirschner B., Griffiths A.M., Katz A.J., Grand R.J., Boyle J.T. (1991). Development and validation of a pediatric Crohn’s disease activity index. J. Pediatr. Gastroenterol. Nutr..

[33] Sýkora J., Pomahačová R., Kreslová M., Cvalínová D., Štych P., Schwarz J. (2018). Current global trends in the incidence of pediatric-onset inflammatory bowel disease. World J. Gastroenterol..

[34] Singh S., Dulai P.S., Zarrinpar A., Ramamoorthy S., Sandborn W.J. (2017). Obesity in IBD: Epidemiology, pathogenesis, disease course and treatment outcomes. Nat. Rev. Gastroenterol. Hepatol..

[35] Sila S., Trivić I., Pavić A.M., Niseteo T., Kolaček S., Hojsak I. (2019). Nutritional status and food intake in pediatric patients with inflammatory bowel disease at diagnosis significantly differs from healthy controls. Eur. J. Pediatr..

[36] Chandrakumar A., Wang A., Grover K., El-Matary W. (2020). Obesity Is More Common in Children Newly Diagnosed with Ulcerative Colitis as Compared to Those with Crohn Disease. J. Pediatr. Gastroenterol. Nutr..

[37] Karaskova E., Velganova-Veghova M., Geryk M., Foltenova H., Kucerova V., Karasek D. (2021). Role of Adipose Tissue in Inflammatory Bowel Disease. Int. J. Mol. Sci..

[38] Dickson I. (2020). Creeping fat in Crohn’s disease explained. Nat. Rev. Gastroenterol. Hepatol..

[39] Schäffler A., Herfarth H. (2005). Creeping fat in Crohn’s disease: Travelling in a creeper lane of research?. Gut.

[40] Harper J.W., Zisman T.L. (2016). Interaction of obesity and inflammatory bowel disease. World J. Gastroenterol..

[41] Kredel L.I., Siegmund B. (2014). Adipose-tissue and intestinal inflammation—Visceral obesity and creeping fat. Front. Immunol..

[42] Krebs N.F., Himes J.H., Jacobson D., Nicklas T.A., Guilday P., Styne D. (2007). Assessment of child and adolescent overweight and obesity. Pediatrics.

[43] Yerushalmy-Feler A., Galai T., Moran-Lev H., Ben-Tov A., Dali-Levy M., Weintraub Y., Amir A., Cohen S. (2021). BMI in the lower and upper quartiles at diagnosis and at 1-year follow-up is significantly associated with higher risk of disease exacerbation in pediatric inflammatory bowel disease. Eur. J. Pediatr..

[44] Selbuz S., Kansu A., Berberoğlu M., Şıklar Z., Kuloğlu Z. (2020). Nutritional status and body composition in children with inflammatory bowel disease: A prospective, controlled, and longitudinal study. Eur. J. Clin. Nutr..

[45] Von Graffenried T., Schoepfer A.M., Rossel J.B., Greuter T., Safroneeva E., Godat S., Henchoz S., Vavricka S.R., Sokollik C., Spalinger J. (2022). Impact of Overweight and Obesity on Disease Outcome in the Pediatric Swiss Inflammatory Bowel Disease Cohort. JPGN Rep..

[46] Kuriyan R. (2018). Body composition techniques. Indian J. Med. Res..

[47] Walters W.A., Xu Z., Knight R. (2014). Meta-analyses of human gut microbes associated with obesity and IBD. FEBS Lett..

[48] Alsharairi N.A. (2022). The Therapeutic Role of Short-Chain Fatty Acids Mediated Very Low-Calorie Ketogenic Diet-Gut Microbiota Relationships in Paediatric Inflammatory Bowel Diseases. Nutrients.

