# Supplementary material for: Disease Activity Is Associated with Obesity in Newly Diagnosed Pediatric Patients with Ulcerative Colitis
Source: Int J Environ Res Public Health. 2022 Dec 1;19(23):16091. doi: 10.3390/ijerph192316091 (PMC9738058; doi:10.3390/ijerph192316091)
Supplement: Supplementary file 1 [file ijerph-19-16091-s001.zip › ijerph-1989856-supplementary.pdf]

International Journal of
Environmental Research and Public Health

MDPI

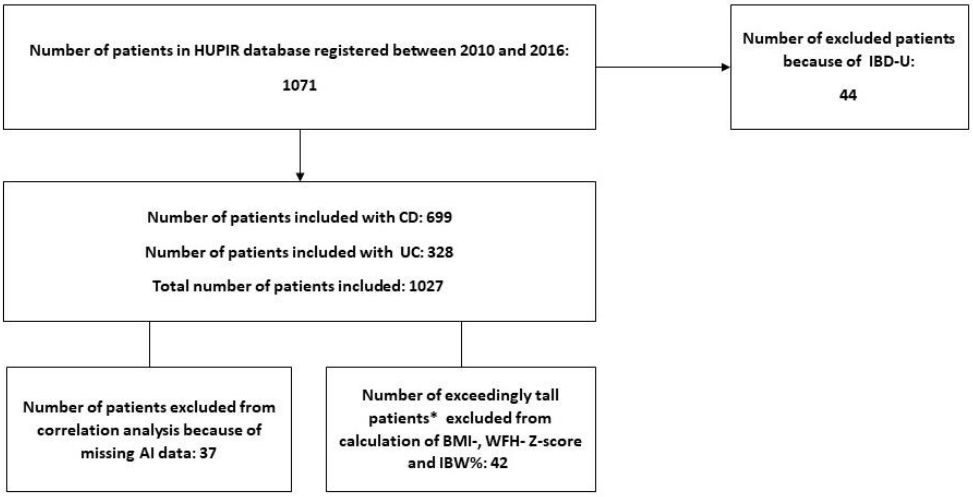

Figure S1. Flowchart of patients. HUPIR: Hungarian Pediatric Inflammatory bowel disease Registry; IBD-U: inflammatory bowel disease unclassified; CD: Crohn's disease; UC: ulcerative colitis, AI: activity index; BMI: body mass index; WFH: weight-for-height; IBW%: ideal body weight percent; * Hungarian reference curves do not contain data above the height of 184 cm in boys and 175 cm in girls.

Table S1. Mean and median value of body weight, BW, BMI, WFH Z-scores and IBW% in patients with CD and UC.

	**CD (N = 699)**	**UC (N = 328)**
**BW Z-score mean (±SD)**	−0.58 (1.07)	−0.15 (1.11)
**BW Z-score median (min–max)**	−0.72(−3.52–4.41)	−0.39 (−3.38–4.40)
**WFH Z-score mean (±SD)**	−0.67 (1.09)	−0.30 (1.18)
**WFH Z-score median (min–max)**	−0.85 (−5.98–5.14)	−0.57 (−2.88–4.26)
**BMI Z-score mean (±SD)**	−0.67 (1.01)	−0.23 (1.18)
**BMI Z-score median (min–max)**	−0.78 (−3.17–4.86)	−0.58 (−2.92–4.05)
**IBW% mean (±SD)**	92.52 (17.31)	98.42 (18.38)
**IBW% median (min–max)**	89.43 (53.95–180.76)	94.06 (60–173.46)

CD: Crohn's disease; UC: Ulcerative colitis; N: number of patients; SD: Standard deviation; BW: body weight, BMI: body mass index, WFH: weight-for-height, IBW%: ideal body weight percent.

Table S2. Mean and median disease activity indices in patients with CD and UC at the time of diagnosis according to different nutritional status.

	PCDAI			PUCAI		
	**N**	**Mean (±SD)**	**Median (min–max)**	**N**	**Mean (±SD)**	**Median (min–max)**
**Body weight**						
Undernourished (Z-score < −2)	33	38.63 (16.57)	35 (10–87.5)	2	57.50 (38.89)	57.5 (30–85)
Normal (Z-score −2–2)	627	30.50 (14.88)	30 (0–85)	290	38.12 (18.71)	35 (5–85)
Obese (Z-score > 2)	17	18.67 (6.91)	20 (10–35)	20	44.25 (20.72)	45 (15–85)
Total	677	30.6 (15.03)	30 (0–87.5)	312	38.64 (19.00)	35 (5–85)
**Body mass index**						
Undernourished (Z-score < −2)	16	45.00 (17.79)	43 (20–87.5)	5	54.44 (9.43)	55 (40–65)
Normal (Z-score −2–2)	401	31.13 (15.30)	30 (0–84)	181	38.32 (19.07)	35 (5–85)
Obese (Z-score > 2)	7	18.92 (8.27)	18 (10–35)	13	45.76 (21.39)	45 (15–85)
Total	424	31.45 (15.59)	30 (0–87.5)	199	39.21 (19.22)	35 (5–85)
**Weight-for-height**						
Undernourished (Z-score < −2)	29	42.15 (16.45)	40 (12.5–87.5)	7	54.60 (15.31)	55 (30–70)
Normal (Z-score −2–2)	602	30.48 (14.89)	30 (0–85)	275	37.63 (18.65)	35 (5–85)
Obese (Z-score > 2)	17	22.94 (9.69)	20 (10–50)	15	44.66 (20.04)	45 (15–80)
Total	648	30.81 (15.08)	30 (0–87)	297	38.38 (18.83)	35 (5–85)
**Ideal body weight percent**						
<70	22	43.29 (14.68)	40 (25–87.5)	2	52.50 (17.67)	53 (40–65)
70–120	580	30.60 (14.86)	30 (0–85)	259	37.74 (18.69)	35 (5–85)
>120	44	27.76 (15.49)	25 (10–84)	36	41.66 (19.19)	40 (10–85)
Total	646	30.84 (15.07)	30 (0–87.5)	297	38.31 (18.77)	35 (5–85)

CD: Crohn's disease; UC: ulcerative colitis; N: number of patients; SD: standard deviation; PCDAI: Pediatric Crohn's Disease Activity Index; PUCAI: Pediatric Ulcerative Colitis Activity Index.

Table S3. Associations between disease activity indices and nutritional status in patients with CD and UC (Pearson analysis).

		**BW Z-score (N)**	**WFH Z-score (N)**	**BMI Z-score (N)**	**IBW% (N)**
**PCDAI**	≤median	R= −0.148 (340)<br>B: −7.440 (−7.542–−1.264)<br>***p* = 0.006**	R= −0.221 (322)<br>B: −7.080 (−10.517–−3.643)<br>***p* = 0.000**	R= −0.314 (212)<br>B: −10.745 (−15.170–−6.320)<br>***p* = 0.000**	R= −0.194 (321)<br>B: −0.465 (−0.724–−0.206)<br>***p* = 0.000**
	>median	R= −0.087 (336)<br>B: −1.468 (−3.282–0.347)<br>*p* = 0.113	R= −0.057 (324)<br>B: −0,888 (−2.600–0.824)<br>*p* = 0.308	R= −0.069 (212)<br>B: −1.194 (−3.534–1.146)<br>*p* = 0.316	R= −0.049 (325)<br>B: −0.047 (−0.152–0.058)<br>*p* = 0.382
**PUCAI**	≤median	R= −0.124 (156)<br>B: −5.489 (−12.484–1.505)<br>*p* = 0.123	R= −0.196 (238)<br>B: −6.955 (−13.981–0.070)<br>***p* = 0.002**	R= −0.164 (101)<br>B: .7.485 (−16.485–1.516)<br>*p* = 0.102	R= −0.174 (150)<br>B: −0.537 (−1.030- −0.044)<br>***p* = 0.033**
	>median	R= 0.143 (156)<br>B: −2.656 (−0.273–5.584)<br>*p* = 0.075	R= 0.234 (58)<br>B: 2.923 (0.286–5.560)<br>*p* = 0.077	R= 0.131 (98)<br>B: −2.977 (−0.405–6.399)<br>*p* = 0.198	R= 0.166 (147)<br>B: 0.171 (0.005–0.337)<br>***p* = 0.044**

PCDAI: Pediatric Crohn's Disease Activity Index; PUCAI: Pediatric Ulcerative Colitis Activity Index; BW: body weight; WFH: weight-for-height; BMI: body mass index; IBW%: ideal body weight percent; N: number of patients, *p*: level of significance ($p < 0.05$), R: correlation coefficient, B: unstandardized regression coefficient (95% confidence interval).
